# Automatic prediction of tumour malignancy in breast cancer with fractal dimension

**DOI:** 10.1098/rsos.160558

**Published:** 2016-12-07

**Authors:** Alan Chan, Jack A. Tuszynski

**Affiliations:** 1 Department of Oncology, University of Alberta, 11560 University Avenue, Edmonton, Alberta, Canada T6G 1Z2; 2 Department of Physics, University of Alberta, Centennial Centre for Interdisciplinary Science, Edmonton, Alberta, Canada T6G 2E1; 3 Department of Mathematical and Statistical Sciences, University of Alberta, Central Academic Building, Edmonton, Alberta, Canada T6G 2G1

**Keywords:** cancer prediction, tumour malignancy, automatic image slide analysis, fractal dimension

## Abstract

Breast cancer is one of the most prevalent types of cancer today in women. The main avenue of diagnosis is through manual examination of histopathology tissue slides. Such a process is often subjective and error-ridden, suffering from both inter- and intraobserver variability. Our objective is to develop an automatic algorithm for analysing histopathology slides free of human subjectivity. Here, we calculate the fractal dimension of images of numerous breast cancer slides, at magnifications of 40×, 100×, 200× and 400×. Using machine learning, specifically, the support vector machine (SVM) method, the F1 score for classification accuracy of the 40× slides was found to be 0.979. Multiclass classification on the 40× slides yielded an accuracy of 0.556. A reduction of the size and scope of the SVM training set gave an average F1 score of 0.964. Taken together, these results show great promise in the use of fractal dimension to predict tumour malignancy.

## 1. Introduction

Exponential increase in technological capability owing to the scope of information technology applications over the past few decades has revolutionized the way modern society functions, especially in terms of communication. The advent of computerization has automated many tasks once seen to be only in the realm of manual labour. At the same time, advances in medical knowledge have allowed us to determine with ever greater precision the particular characteristics of the ailments that afflict us, as well as improved treatments to ameliorate our lives. The diagnosis of cancer is one area of medicine that isamenable to automation but so far has been largely dependent on the traditional approaches, using the expertise of trained professionals.

Histological evaluation of a tissue sample is a critical step in the diagnosis of cancer, providing important prognostic information [1,2]. However, significant inter- and intravariability exists between pathologists [3,4], especially in ‘borderline’ cases [5]. Indeed, different diagnoses can drastically change the treatment options chosen for patients and substantially affect the outcomes [4]. Therefore, it is clearly of significance to maximize the reliability of histological evaluation to be more in line with the use of computerized methods that have improved the quality in many areas of technology, including medical imaging. There are at least two ways in which to improve histological evaluation: the development of a quantitative measure sufficiently well-linked to cancer severity and the use of an automated program to make objective calculations of such a measure. This paper intends to propose some algorithms that can be used in this connection.

## 2. Background

### 2.1. Past work

There have been many attempts to automate the diagnosis of cancer from histology slides. Common to all is a three-step process: (i) preprocessing of the image, (ii) extraction of relevant features, and (iii) diagnosis from those features [6]. The step of greatest interest, with perhaps the greatest diversity in methods, is the feature extraction step. The goal here is to select features of an image that may be amenable to quantification and subsequent computational analysis, while at the same time being good measures of cancer severity. That is, the hope is to correlate an image feature with either a diagnostic or a prognostic indicator, including, but not limited to, tumour malignancy and a related survival rate.

A plethora of features have been proposed and tested in the context of histopathology of cancer, namely fractal dimension [7–10], entropy [11], textural features based on the image histogram [12], and even features that were automatically selected by an unsupervised machine learning algorithm [13]. Often, a large number of features are used at the same time [14,15]. In this paper, we focus on further testing of fractal dimension as a viable image feature that can lead to a high level of confidence in the resultant classification. We extend the work done previously on this particular feature by testing on a larger dataset that has been selected for its consistency and quality, applying machine learning for prediction of tumour malignancy, and running further classification procedures to increase our confidence in the extent to which our hypothesis may be generalized.

### 2.2. Fractal dimension

To best understand what fractal dimension is, it is helpful to start with the familiar notion of topological dimension in geometry. A line has topological dimension 1, a square has topological dimension 2 and a cube has topological dimension 3. Intuitively, topological dimension is a measure of how everyday, regular objects change with scaling, and is usually an integral value. A metre stick of length 1 m is required to measure a 1 m line. If we use a half-metre stick, then double the number sticks will be required to measure the line. Similarly, we may use a square of side length 1 m to measure the same square of side length 1 m. If we halve the length of the measuring square, then we now require four squares to measure the full 1 m × 1 m square. More concretely,

2.1 $$N=\epsilon^{-d},$$

where *N* is the number of sticks required, *ϵ* is the scaling factor and *d* is the topological dimension. Solving for *d*,

2.2 $$d=-\frac{\log(N)}{\log(\epsilon )}.$$

**Figure 1.**
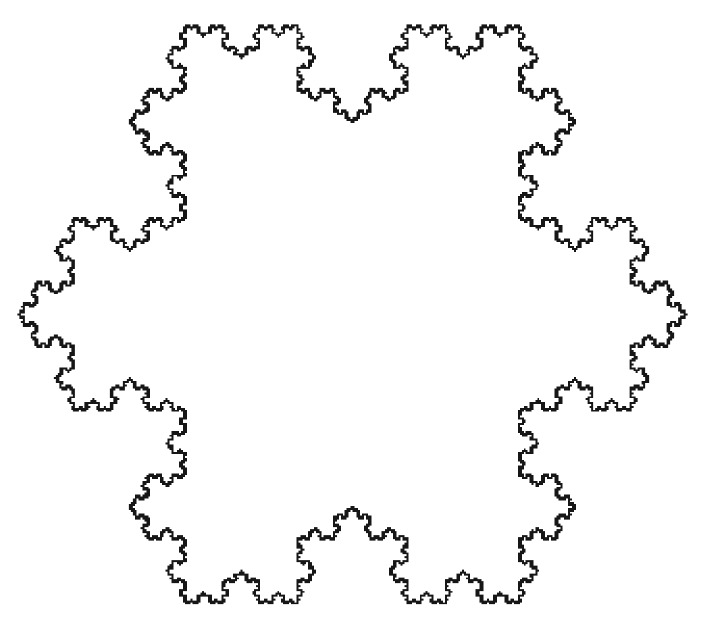
An image of the Koch snowflake, a fractal with fractal dimension *d*≈ 1.26. From https://commons.wikimedia.org/wiki/File:Flocke.PNG, licensed under Creative Commons.

Regarding our square example, we have d=−log⁡(4)/(log⁡(1/2))=2 which is what we expect the dimension of a two-dimensional image to be. In figure 1, we show an example of a geometrical fractal which is called the Koch snowflake whose fractal dimension is approximately 1.26.

However, nature often does not conform to the pristine regularity of Euclidean geometry. In particular, there are objects whose dimension is not integral, but real-valued, accompanied by unexpected scaling behaviour. We label such d∈R the fractal dimension. Qualitatively, non-integral fractal dimension may be visually recognizable by a more complex image border, and self-similarity after scaling. Indeed, the ‘fractal’ of fractal dimension refers to the often unusual scaling behaviour of fractal objects, which may be both geometrical and physical. The former are created by applying an iterative rule to a motif while the latter are created by a physical process such as diffusion-limited aggregation or a biological process such as tumour growth, for example.

In fact, the fractal dimension of any image on a two-dimensional surface will always satisfy *d*∈[1,2]. That is, the dimension of the image is bounded above by 2, the dimension of the containing environment, and bounded below by 1, the dimension of any closed curve on the two-dimensional surface.

The obvious question is how one goes about calculating fractal dimension. In this paper, we use the box-counting algorithm. The procedure is described in great detail in [16], but we briefly explain it here for the reader’s benefit.

A series of boxes of side length *ϵ* are fitted, without overlap, over the image to be analysed. The number of boxes that contain some portion of the image are then counted. The logarithm of that number is divided by the negative logarithm of the size of the boxes used. We, therefore, have

2.3 $$d_{b}=\lim_{\epsilon \to 0}-\frac{\log(N(\epsilon ))}{\log(\epsilon )}=\lim_{\epsilon \to 0}\frac{\log(N(\epsilon ))}{\log(1/\epsilon )},$$

where *N*(*ϵ*) denotes the number of non-empty boxes of side length *ϵ*. There is a clear connection between this formula and that of the original definition of fractal dimension. Because we cannot apply a limit operation to images that do not have an infinite precision in detail, we instead calculate −log⁡(N(ϵ))/log⁡(ϵ) for various values of *ϵ*, and find the slope of log⁡(N(ϵ)) plotted against log⁡(1/ϵ).

We have mentioned the Koch fractal only as an example. Its fractal dimension of 1.26 is relatively low, as will be shown below, by comparison with the fractal dimensions of the pathology slides analysed here. Another type of geometrical fractal, the so-called Cesaro curve, may be a closer geometrical approximation to pathology images, owing to its fractal dimension which is close to 2.0 depending on the angle between the line segments in its motif. More information about fractals and their properties may be found in [17].

## 3. Data

In order to examine the viability of using the fractal dimension for histopathology of breast cancer, we have decided to use the BreaKHIS database in this study. This database has a high degree of consistency and image quality that was important in making the subsequent results free of artefacts. It is a sufficiently large dataset to allow for statistical significance because it consists of 7909 breast cancer histopathology images, acquired from 82 patients by [18], publicly available from http://web.inf.ufpr.br/vri/breast-cancer-database. It is a freely available Web-based dataset, and we encourage the interested reader to access these data for inspection. Images were collected through a clinical study from January 2014 to December 2014. Samples were taken from breast tissue biopsy slides, and are all stained with haematoxylin and eosin (H&E). Owing to the internal consistency of the images, we were able to avoid dealing with such common problems as image artefacts, e.g. colour batch effects. The BreaKHIS dataset includes both benign and malignant images, at magnifications of 40×, 100×, 200× and 400×. A tally is in table 1. Each image is 700×460 pixels, and is of the PNG image file format.

**Table 1.** The distribution of images in BreaKHIS, from [18].

magnification	benign	malignant	total
40×	625	1370	1995
100×	644	1437	2081
200×	623	1390	2013
400×	588	1232	1820
total	2480	5429	7909
no. patients	24	58	82

**Figure 2.**
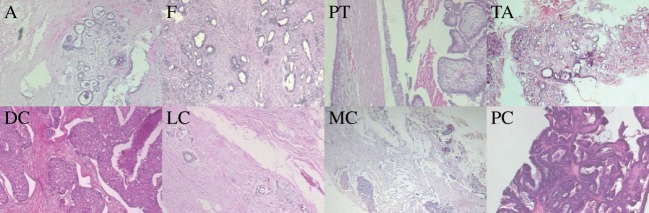
Representative benign and malignant images at 40× magnification. Benign: adenosis (A), fibroadenoma (F), phyllodes tumour (PT) and tubular adenoma (TA). Malignant: ductal carcinoma (DC), lobular carcinoma (LC), mucinous carcinoma (MC) and papillary carcinoma (PC).

Furthermore, the benign and malignant images are split into subtypes. The benign tumour images contain slides of adenosis (A), fibroadenoma (F), phyllodes tumour (PT) and tubular adenoma (TA). The malignant tumour images contain slides of ductal carcinoma (DC), lobular carcinoma (LC), mucinous carcinoma (MC) and papillary carcinoma (PC). In figure 2, we show representative images of each subtype. In tables 1–3, we summarize the distribution of images in the BreaKHIS database for benign and malignant cases as well as into the above subtypes.

**Table 2.** The distribution of images of benign tumours in BreaKHIS, from [18].

magnification	A	F	TA	PT	total
40×	114	253	109	149	625
100×	113	260	121	150	644
200×	111	264	108	140	623
400×	106	237	115	130	588
total	444	1014	453	569	2368
no. patients	4	10	3	7	24

**Table 3.** The distribution of images of malignant tumours in BreaKHIS, from [18].

magnification	DC	LC	MC	PC	total
40×	864	156	205	145	1370
100×	903	170	222	142	1437
200×	896	163	196	135	1390
400×	788	137	169	138	1232
total	3451	626	792	560	5429
no. patients	38	5	9	6	58

## 4. Method

Mathematica was the programming environment used in this study. Fortunately, this programming tool has an edge detection tool that allows one to find sharp boundaries between different areas of an image. First, fractal dimensions for all 7909 images were calculated. This process was accomplished in several steps described below.

1. Binarize all images and detect the edges, with Mathematica's built-in functions *Binarize* and *EdgeDetect*.
2. Calculate the integral image of each image. The process is detailed in [19]. The calculation of the integral image is not necessary, but vastly speeds up the algorithm by allowing quick calculation of the number of pixels in a given box.
3. We take *ϵ* from three pixels to four pixels, with a step size of only 1. While this decision gives only two data points, the results of our study justify our choice. Furthermore, the usage of larger side lengths would have introduced misleading values for the fractal dimension into our calculations.
4. For each *ϵ*, the image is partitioned into non-overlapping boxes of side length *ϵ*. The number of pixels in a given box is calculated from the integral image. The total number of non-empty boxes is summed, and put into an ordered pair with 1/*ϵ*. We then take the logarithm of both values.
5. The fractal dimension of each image is calculated by fitting a straight line to the just-calculated data points of each image, and recording the slope of the line as the fractal dimension of the image. The Mathematica function *Fit* was used in this step.

**Figure 3.**
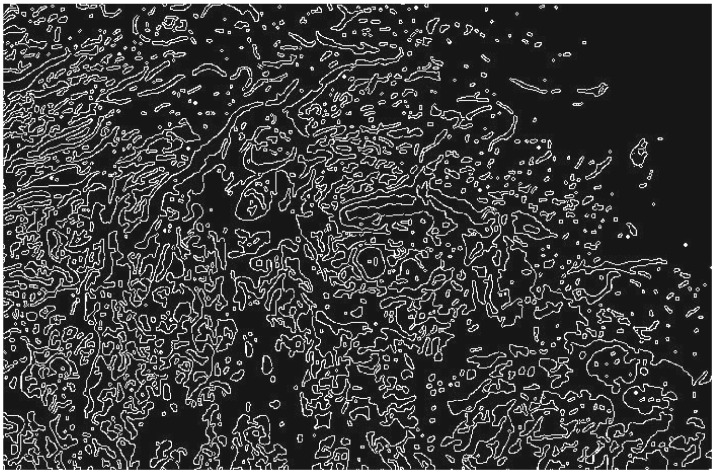
Image of a 40× slide of ductal carcinoma, after binarization and edge detection.

Without loss of generality, we describe the procedure taken with the 40× slides. Figure 3 is an example that can be used as a reference. Similar steps were taken in the remaining cases. We used a randomly selected set consisting of 50% of the benign tumour fractal dimensions and a randomly selected set consisting of 50% of the malignant tumour fractal dimension as a training set, setting aside the rest of the data for use as a validation set. We trained a support vector machine (SVM) algorithm, on the training set, to classify whether a tumour was benign or malignant based solely on fractal dimension. Strictly speaking, because we had only one image feature, an SVM was not absolutely necessary. Indeed, a cut-off fractal dimension value could have been found by hand. However, in the interests of precision and certainty, we chose to use an SVM. Obviously, other machine-learning methods can be used here and will most likely be equally successful in their performance. Moreover, with only one feature used, one could indeed resort to classical linear discriminant analysis between clouds of points using for example Mahalanobis distance [20] to replace a somewhat arbitrary determination of the cut-off dimension.

From the results of the SVM predictions on the validation set, we decided whether or not to continue with further classification trials. Further trials were only pursued with the 40× slides.

A multiclass classification was then attempted. Instead of classifying the 40× slides by benign versus malignant, we tried to classify the images based on the eight subtypes of benign and malignant tumours. That is, we predicted the tumour subtype to which a given tissue image belonged. A random half of the fractal dimensions of each subtype served as a training set.

Afterwards, we performed another classification trial, this time modifying the size of the training set. In total, 16 different SVMs were trained, each using a different training set. For each SVM, the training set consisted of the fractal dimensions of a single benign subtype, and a single malignant subtype. Because there are four benign subtypes and four malignant subtypes, we have 16 different possible combinations for the training sets, and hence 16 SVMs. The SVMs were then tested against the fractal dimensions upon which they were not trained. For example, an SVM was trained against adenoma and ductal carcinoma, and subsequently tested against fibroadenoma, phyllodes tumour, tubular adenoma, lobular carcinoma, mucinous carcinoma and papillary carcinoma.

## 5. Results

**Figure 4.**
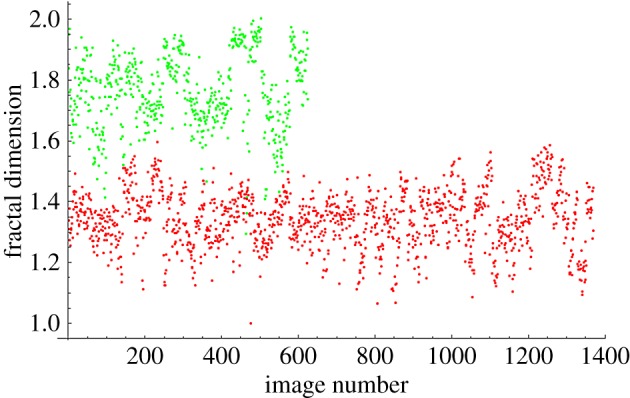
Fractal dimensions for 40× images.

**Figure 5.**
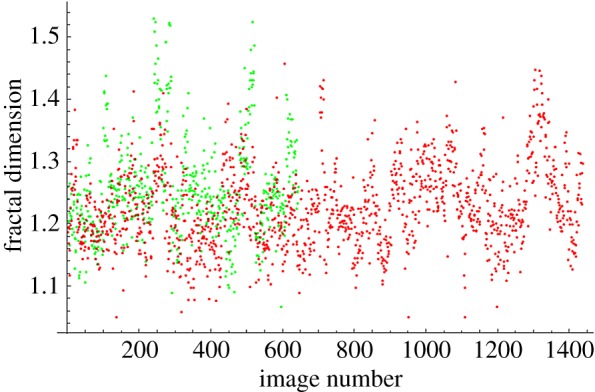
Fractal dimensions for 100× images.

**Figure 6.**
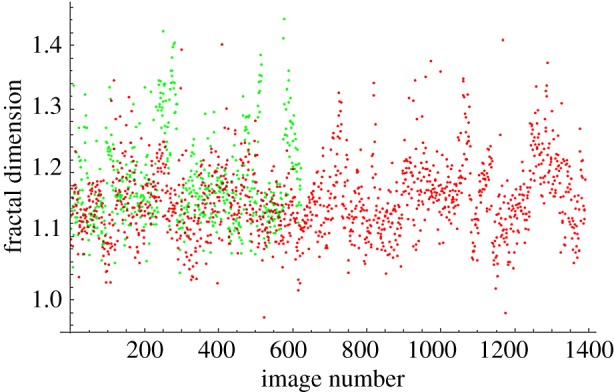
Fractal dimensions for 200× images.

**Figure 7.**
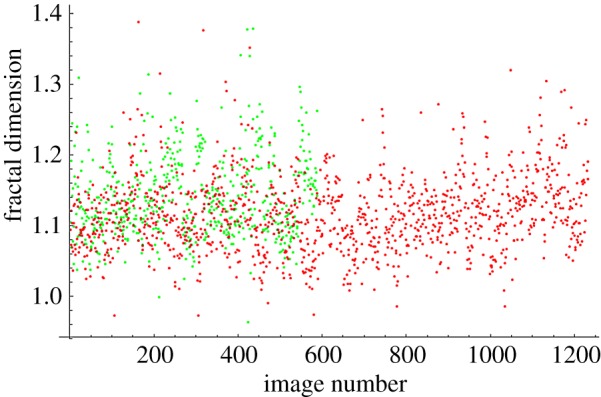
Fractal dimensions for 400× images.

The fractal dimensions for all images at each magnification are plotted in figures 4–7. The green dots represent benign images, and the red dots represent malignant images. The *y*-axis corresponds to fractal dimension, and the *x*-axis corresponds to the arbitrary image numbering.

The results for the SVM classification of benign versus malignant, with a randomized training set, are shown in tables 4 and 5. We calculated the true positive rate (TP), the true negative rate (TN) and the F1 score, otherwise known as the harmonic mean, of TP and TN. True positives are where the SVM correctly classified an image as malignant, and true negatives are where the SVM correctly classified an image as benign. TP and TN are calculated as ratios between 0 and 1, rounded to three decimal digits. Note that to calculate the F1 score, we have used the formula: *F*1=(2×*TP*×*TN*)/(*TP*+*TN*). This harmonic average formula was used for simplicity and convenience. A different type of averaging can also be applied.

**Table 4.** Classification accuracy for benign versus malignant images at all magnifications.

magnification	TP	TN	F1
40×	0.990	0.968	0.979
100×	0.983	0.090	0.165
200×	0.974	0.090	0.165
400×	0.940	0.146	0.253

**Table 5.** Benign versus malignant classification accuracy with different training sets.

BT	MT	TP	TN	F1
A	DC	0.988	0.953	0.970
A	LC	0.993	0.957	0.975
A	MC	0.979	0.967	0.973
A	PC	0.999	0.945	0.971
F	DC	0.982	0.930	0.955
F	LC	0.975	0.946	0.960
F	MC	0.995	0.922	0.957
F	PC	0.999	0.901	0.947
PT	DC	1.000	0.913	0.956
PT	LC	0.993	0.942	0.967
PT	MC	0.999	0.913	0.954
PT	PC	1.000	0.895	0.945
TA	DC	0.992	0.956	0.974
TA	LC	0.974	0.975	0.974
TA	MC	0.994	0.960	0.977
TA	PC	0.999	0.954	0.976
mean				0.964

Based on the results in table 4, we chose only to pursue further testing with the 40× fractal dimensions, which gave us the best predictive power. We created another training set containing a random half of the images of each tumour subtype. Another SVM was trained on this set, and had to classify the rest of the images based on subtype. Accuracy, the number of correct predictions divided by the total number of samples, was 0.556.

Sixteen different SVMs were then trained, with each training set consisting of the fractal dimensions of a single benign (BT) and single malignant subtype (MT). The SVM then tried to classify the remaining subtypes by malignancy. As before, we calculated the TN, TP and F1 scores. We re-use the abbreviations for the subtypes, repeated here: adenosis (A), fibroadenoma (F), phyllodes tumour (PT) and tubular adenoma (TA). The malignant tumour images contain slides of ductal carcinoma (DC), lobular carcinoma (LC), mucinous carcinoma (C) and papillary carcinoma (PC). The resulting accuracies are in table 5.

## 6. Discussion

The plots shown in figures 4–7 show visually the results of table 4. It is clear that with just fractal dimension as the sole feature, the best hope for diagnostic application is through examination of 40× slides. A possible explanation is that as one increases the magnification, it becomes more difficult to see higher-level features of the cell morphology that are often indicative of cancer, like poor cell differentiation, and the additional details presented by these higher-resolution images are actually making diagnostic interpretation more difficult. To quote an old adage: ‘it is hard to see the forest for the trees’. It is notable that an F1 score of 0.979 was obtained for the 40× slides, with the use of only the one image feature of fractal dimension making this a very powerful yet simple process. This is consistent with the numerous studies in the past that linked a change in the fractal dimension with the initiation and progression of cancer [21–23].

It is interesting to note that in Tambasco’s study [7], the fractal dimension is directly correlated with tumour grade. That is, for higher values of fractal dimension, a higher tumour grade was found, from which can be deduced that the tumour was probably more malignant. However, our study found the opposite relationship, that higher values of fractal dimension tend to be associated with lower tumour malignancy. A possible reason is the different stains that may have been used in obtaining histopathology images. The Tambasco study used a pan-keratin stain, which tends to exclude stromal cells and other extracellular features from the tissue slides [7]. The tissue slides from BreaKHIS were stained with H&E [18], a common stain for histology specimens. The inclusion of extracellular features in the H&E stain could have resulted in an increased fractal dimension of the images. Indeed, if fractal dimension is an intuitive measure of complexity, then it seems natural that the complexity of a benign sample is higher than that of a malignant sample probably owing to proper cell differentiation and hence the development of specific morphological features characteristic of a given cell type.

The use of fractal dimension to classify subtypes as the only feature had an accuracy of 0.556. Likely, there is not enough information contained in fractal dimension to distinguish between different subtypes of benign slides or malignant slides. More features would be required to effectively classify subtypes.

According to the data in table 5, the use of different training sets for the SVM did not much affect the accuracy of predictions. The average F1 score across all rows in table 5 was 0.964, close to the value in table 4 of 0.979. There are two implications. The first is the existence of great similarity in fractal dimension among benign and malignant tumours. The second is the capacity of fractal dimension to be generalized to unseen subtypes. Accuracy of the SVM was not severely affected by limiting the training set to a certain subtype. Indeed, it seems that the only significant limiting factor was the size of the training set. Therefore, it is reasonable to suspect that fractal dimension may also be a precise measure of malignancy in benign and malignant subtypes that were not present in the BreaKHIS dataset.

## 7. Conclusion

The use of fractal dimension to classify tumours based on malignancy shows great potential for practical applications in pathological analysis of various types and subtypes of cancer. Here, we have only used it for several subtypes of breast cancer. At the magnification of 40×, the F1 score was 0.979, using an SVM trained on a random half of the BreaKHIS dataset. Multiclass classification seems to require more features, with an accuracy of just 0.556. However, the ability of fractal dimension to distinguish generally between benign and malignant slides seems formidable, given that reduction in the size and scope of the training set had little effect on the classification accuracy, which was an average of 0.964.

Further data, including different subtypes and different cancers, should be used to verify the hypothesis. In particular, it is important to emphasize that the images produced are stain-dependent, and one must ensure uniformity and quality control in order to be able to produce reliable results. Our dataset did not include normal tissue as control and this should be remedied in the future. In addition, we have not found information about the inclusion of tumour stroma in the images, so the issue of stroma-poor or stroma-rich samples should be addressed separately in a future study involving other datasets. In addition, this method should be applied prospectively to truly determine its limitations in a blind experiment, as opposed to the retrospective analysis performed here. It would also be interesting to find a correlation between the fractal dimension of the pathology slide and a clinical outcome measure such as 5 year survival of the patient.

However, the usage of a simple, single image feature to predict tumour malignancy holds great promise in increasing the reliability of pathological diagnoses and the speed with which the biological data are analysed. At the very least, this could be used to assist in the diagnostic procedures and reduce the time burden on pathologists who routinely deal with large numbers of patient samples and who are prone to both fatigue and human error with the significant consequences for patient outcomes.

## Data accessibility

The data used in this paper are publicly available and can be accessed online using the BreaKHIS database: http://web.inf.ufpr.br/vri/breast-cancer-database.
